# US Population Eligibility and Estimated Impact of Tirzepatide Treatment on Obesity Prevalence and Cardiovascular Disease Events

**DOI:** 10.1007/s10557-024-07583-z

**Published:** 2024-06-08

**Authors:** Nathan D. Wong, Hridhay Karthikeyan, Wenjun Fan

**Affiliations:** 1 Heart Disease Prevention Program, Division of Cardiology, University of California, Irvine, Irvine, USA; 2 Department of Epidemiology and Biostatistics, University of California, Irvine, Irvine, USA

**Keywords:** Obesity, Cardiovascular disease, Prevention, Tirzepatide, Glucagon-like peptide-1 (GLP-1), Glucose-dependent insulinotropic peptide (GIP)

## Abstract

**Purpose:**

Tirzepatide promotes weight loss and reduces risk factors for cardiovascular disease (CVD) in adults with overweight and obesity. We examined the number of US adults eligible for tirzepatide and its impact on obesity and CVD events.

**Methods:**

We identified US adults aged ≥ 18 years from the cross-sectional US National Health and Nutrition Examination Survey (NHANES) 2015–2018 eligible for tirzepatide based on SURMOUNT-1 trial eligibility criteria. Weight changes in SURMOUNT-1 from tirzepatide 15 mg treatment were used to project the impact on weight change and obesity prevalence in the population assuming titration to this dosage. We estimated 10-year CVD risks from BMI-based Framingham CVD risk scores before and after applying tirzepatide 15 mg treatment BMI and risk factor effects from SURMOUNT-1, the differences in estimated risks multiplied by the eligible NHANES weighted population representing the estimated “preventable” CVD events.

**Results:**

We identified 4015 US adults (estimated population size of 93.4 million [M]) to fit SURMOUNT-1 eligibility criteria, representing 38% of US adults. When the effects of 15 mg tirzepatide were applied, we estimated 70.6% (65.9 M) and 56.7% (53.0 M) of adults to show ≥ 15% and ≥ 20% reductions in weight, respectively, translating to 58.8% (55.0 M) fewer persons with obesity. Among those without CVD, estimated 10-year CVD risks were 10.1% “before” and 7.7% “after” tirzepatide “treatment” reflecting a 2.4% absolute (and 23.6% relative) risk reduction translating to 2.0 million preventable CVD events over 10 years.

**Conclusion:**

Tirzepatide treatment in appropriate US adults may substantially reduce obesity prevalence and CVD events, impacting beneficially on associated healthcare costs.

**Graphical Abstract:**

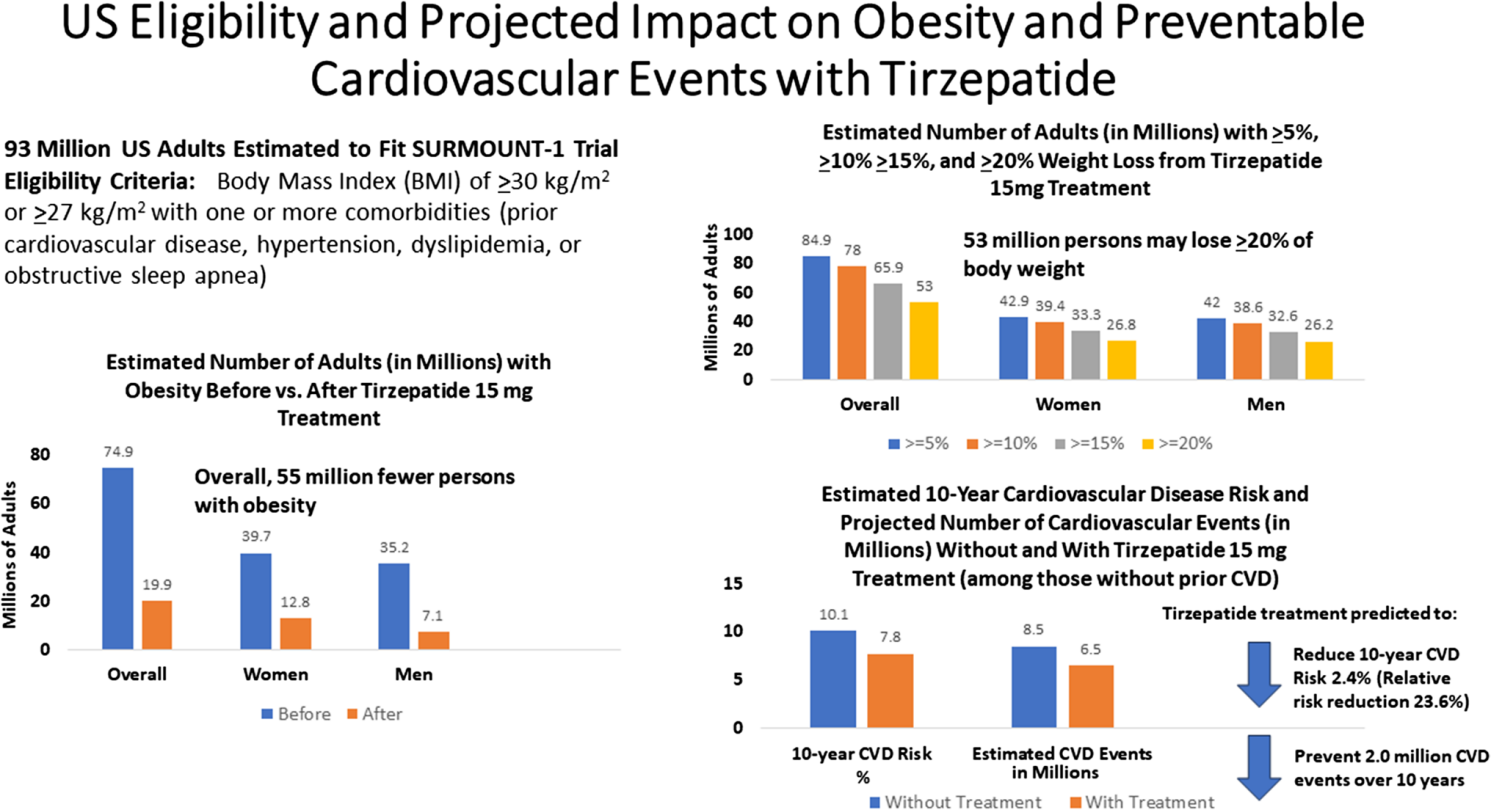

**Supplementary Information:**

The online version contains supplementary material available at 10.1007/s10557-024-07583-z.

More than 40% of the US adult population has obesity, and nearly three-fourths are either overweight or obese [1]. Major disparities are present by racial and ethnic groups, with non-Hispanic Black females having the highest prevalence of obesity (56.8%) [2]. By the end of this decade, half of US adults are expected to have obesity and nearly a fourth will have severe obesity [3]. Moreover, the importance of obesity as a risk factor for cardiovascular disease (CVD) is well-established [4, 5], warranting the need to further demonstrating the value of weight loss interventions on reducing CVD outcomes.

It is well-established that certain glucagon like peptide-1 (GLP-1) receptor agonists reduce CVD events in persons with diabetes [6, 7]. Recently, however, GLP-1 receptor agonists liraglutide [8] and semaglutide [9] in dosages greater than those used for diabetes provide substantial body weight loss and have generated significant excitement in these and other weight loss therapeutics. Tirzepatide is the first-of-class dual glucose-dependent insulinotropic polypeptide (GIP)—GLP-1 receptor agonist, which include the satiety effects of GLP-1 signaling together with the effects of glucagon increasing energy expenditure leading to potentially greater weight loss than GLP-1 agonism alone [10], The SURMOUNT-1 trial recently demonstrated the efficacy of once weekly tirzepatide in adults who are overweight or with obesity to result in up to 20.9% reductions in body weight with 15 mg dosages [11]. In addition, there were also reductions in blood pressure, fasting plasma glucose, and cholesterol levels in those on tirzepatide.

Important to understanding the population impact of these newer therapies is to identify the eligible US population that could benefit from them, as well as their potential impact on the prevalence of overweight/obesity and CVD outcomes. We aim in this study to (1) estimate the eligible US population for tirzepatide based on the SURMOUNT-1 trial eligibility criteria, (2) project the US population impact of tirzepatide on weight loss and obesity prevalence, and (3) estimate the preventable CVD events based on the CVD risk factor effects from the SURMOUNT-1 trial. Having this information may be helpful to better understand the potential CVD benefits of tirzepatide as well as the implications of the ongoing tirzepatide SURMOUNT-MMO cardiovascular outcomes trial [12].

## Materials and Methods

**Fig. 1:**
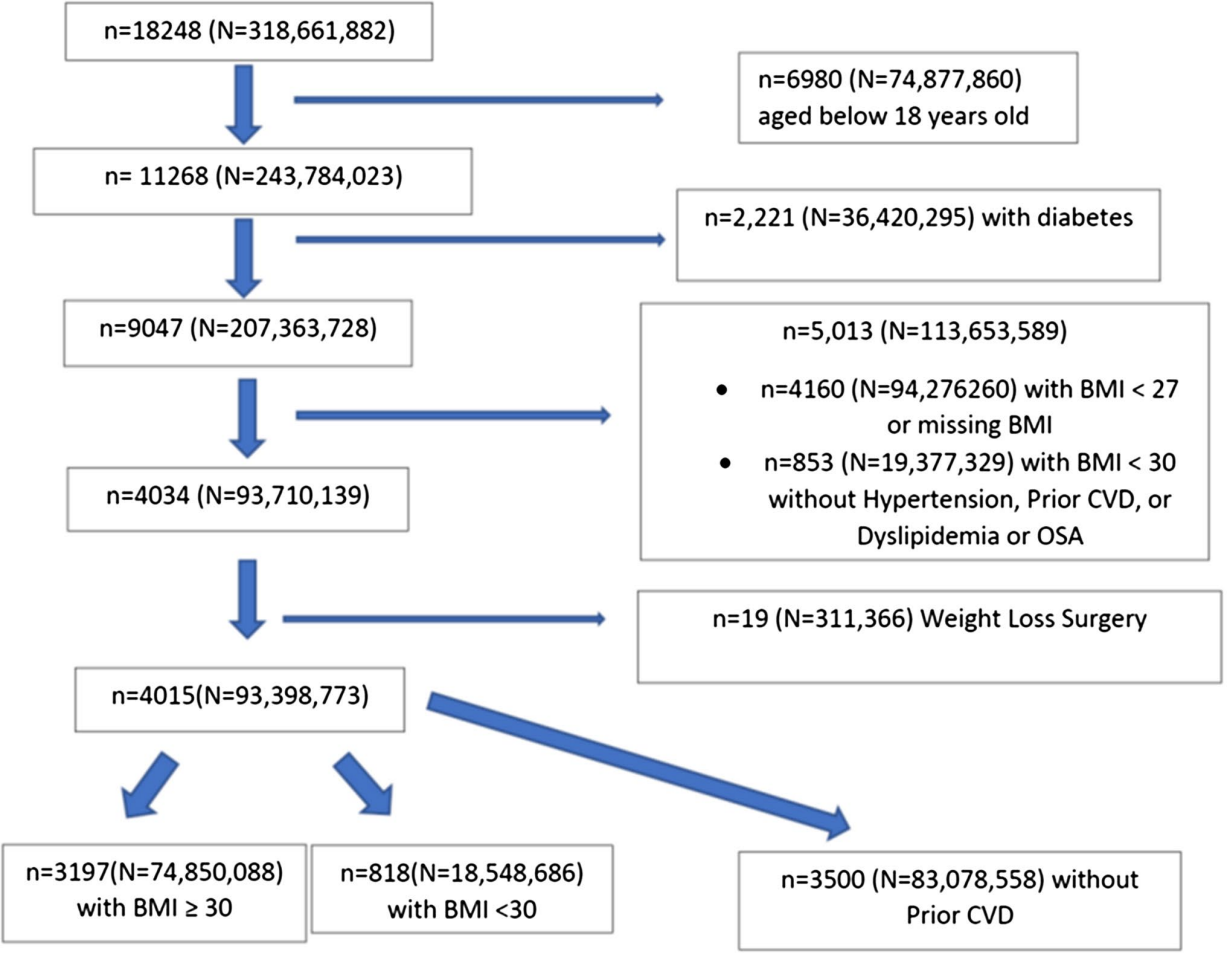
SURMOUNT-1 Eligible Sample Selection from the National Health and Nutrition Examination Surveys 2015–2018. *n* represents sample size; *N* represents population-weighted sample size

We analyzed data from the US National Health and Nutrition Examination Surveys (NHANES) 2015–2018 where subjects provided informed consent to participate. The current study involved the use of publicly available de-identified data and was exempt from institution review board review. We applied eligibility criteria from the tirzepatide SURMOUNT-1 trial [11] to obtain our sample for this study (Fig. 1). Participants were adults aged ≥ 18 years with either a body mass index (BMI) of ≥ 30 kg/m^2^ or a BMI of ≥ 27 kg/m^2^ with at least one or more of the following weight-related conditions: prior CVD, hypertension, dyslipidemia, or obstructive sleep apnea (Central Illustration). We defined hypertension as a blood pressure (BP) ≥ 130/80 mmHg or on anti-hypertensive medication, dyslipidemia as a total cholesterol ≥ 200 mg/dL or triglycerides ≥ 200 mg/dL, or on lipid lowering medication, and prior CVD as a self-reported history of coronary heart disease, heart attack, stroke, or heart failure. The criteria for obstructive sleep apnea in NHANES included an occasional or frequent snore, snort, or stopping breathing while asleep at least 3 times a week. Persons were not eligible for our study if they had (1) a history of diabetes mellitus (DM) defined by a HbA1c of 6.5% or greater, fasting glucose ≥ 126 mg/dL, non-fasting glucose ≥ 200 mg/dL, or doctor told have DM, or taking DM medication or insulin and (2) a history of surgical obesity treatment.

We used NHANES sample weighting to project our sample to the US population in millions who would be eligible for tirzepatide based on the SURMOUNT-1 trial criteria and who are in the overweight (BMI ≥ 27 kg/m^2^ with known risks as described above) or with obesity (BMI of ≥ 30 kg/m^2^), overall and by sex and ethnicity.

We estimated the number of US adults, in millions, who could be projected to achieve weight reductions of ≥ 5%, ≥ 10%, ≥ 15%, and ≥ 20% based on the observed reductions in weight from the tirzepatide 15 mg group in the SURMOUNT-1 trial [9]. We utilized the treatment benefits observed in the 15 mg group to demonstrate what the maximum benefits would be if all eligible patients were titrated to this dosage. We also calculated changes in obesity and overweight prevalence after applying these weight reductions to our study sample. Analyses were additionally stratified by sex and ethnicity utilizing overall trial effects in the 15-mg group, since sex and ethnic-specific results were not available.

We further calculated the 10-year CVD event risk from by D’Agostino and colleagues utilizing the BMI-based Cox regression equations [13] (Appendix) using the CVD risk factor levels in our identified NHANES sample. We then applied the risk factor changes from the SURMOUNT-1 trial tirzepatide 15 mg group (as shown in Table S4 in the Appendix of the SURMOUNT-1 publication [11]) to estimate the “post-treatment” 10-year CVD risk similarly with these equations. These risk factor changes included 20.9% for weight and − 7.6 mmHg (subsequently translated to a percentage change which was then applied to the sample) for systolic blood pressure (SBP) for these risk calculations. We then multiplied this risk by our eligible population size to estimate the number of CVD events without tirzepatide (baseline level) and with tirzepatide (post-treatment), with the difference between the two representing the preventable CVD events that can be projected from tirzepatide treatment. These analyses were stratified by sex and ethnicity. Since weight reduction was our primary analyses involved the use of the BMI-based model as described above, and given that weight reduction was the primary endpoint in the SURMOUNT-1 trial, a sensitivity analysis was done using the laboratory-based equations [13] (Appendix) and the respective SURMOUNT-1 trial effects on total and high-density lipoprotein-cholesterol (HDL-C) (of − 7.4% and + 8.2%, respectively) instead of BMI. SAS version 9.4 was used for our analyses.

## Results

Among 18,248 participants in the NHANES 2015–2018, we identified 4015 (projected to 93.4 M) who fit SURMOUNT-1 trial inclusions and were included in our study. We first examined demographic and risk factor characteristics obtained from our NHANES sample and presented these together with placebo group data from the SURMOUNT-1 trial for comparison (Table 1). Mean age differed slightly between the SURMOUNT-1 trial and our NHANES sample (44.4 vs. 47.3 years), with our study sample having a smaller percentage of female, White, Hispanic/Latino, and Asian race/ethnicity, while a greater proportion were Black. Our NHANES sample also had proportionately more persons with pre-diabetes and greater average low-density lipoprotein cholesterol (LDL-C), higher HDL-C, but lower triglycerides, diastolic blood pressure (DBP), BMI, and proportions of participants with class II (BMI 35 – < 40 kg/m^2^) and class III (BMI ≥ 40 kg/m^2^) obesity compared to the SURMOUNT-1 group.

**Table 1:** Descriptive statistics among SURMOUNT-1 trial and NHANES sample participants

Characteristic	SURMOUNT-1 trial placebo (*n* = 643) Mean ± SD	NHANES sample (*n* = 4015) 93.4 M Mean ± SEM
Age — years	44.4 ± 12.5	47.3 ± 0.5
Female sex — no. (%)	436 (67.8)	2172 (50.5, 47.2 M)
Race or ethnic group — no. (%)*		
Asian	71 (11.0)	233 (2.6, 2.41 M)
Black or African American	55 (8.6)	974 (12.0, 11.1 M)
White	450 (70.0)	1405 (63.3, 59.1 M)
Other	67 (10.4)	204 (5.0, 4.6 M)
Hispanic or Latino — no. (%)	310 (48.2)	1199 (17.2, 16.1 M)
Body weight — kg	104.8 ± 21.4	97.8 ± 0.5
Mean body mass index	38.2 ± 6.9	34.4 ± 0.2
Body mass index category — no. (%)		
< 30	24 (3.7)	818 (19.9, 18.5 M)
≥ 30 to < 35	227 (35.3)	1768 (44.8, 41.8 M)
≥ 35 to < 40	180 (28.0)	819 (20.5, 19.2 M)
≥ 40	212 (33.0)	610 (14.8, 13.9 M)
Waist circumference — cm	114.0 ± 14.9	111.4 ± 0.4
Blood pressure — mm Hg		
Systolic	122.9 ± 12.8	125.5 ± 0.4
Diastolic	79.6 ± 8.0	73.1 ± 0.4
Pulse — beats/min	72.9 ± 9.3	72.9 ± 0.3
Lipid levels — geometric mean mg/dl (coefficient of variation)		
Total cholesterol	186.4 (20.3)	195.2 (20.7)
HDL cholesterol	46.5 (26.9)	50.8 (28.5)
LDL cholesterol	108.4 (30.5)	117.5 (29.2)
Triglycerides	130.5 (49.2)	123.7 (83.5)
Estimated GFR — ml/min/1.73 m^2^	98.1 ± 18.3	94.9 ± 0.7
Prediabetes, *n* (%)	270 (42.0)	2011 (47.5, 44.4 M)
Glycated hemoglobin — %	5.6 ± 0.4	5.49 ± 0.008
Fasting glucose — mg/dl	95.7 ± 9.5	102.9 ± 0.5
Fasting insulin — mlU/liter	14.3 ± 9.9	15.6 ± 0.3

^*^Data for race from the SURMOUNT-1 trial included Hispanic Whites, Asians, Blacks, and Other, with a non-mutually exclusive category for all Hispanics. The NHANES sample included non-Hispanic White, Asian, Black, and other persons, with a mutually exclusive category for Hispanic/Latino. Sample sizes (*n*) presented with weighted population in millions (M)

**Fig. 2:**
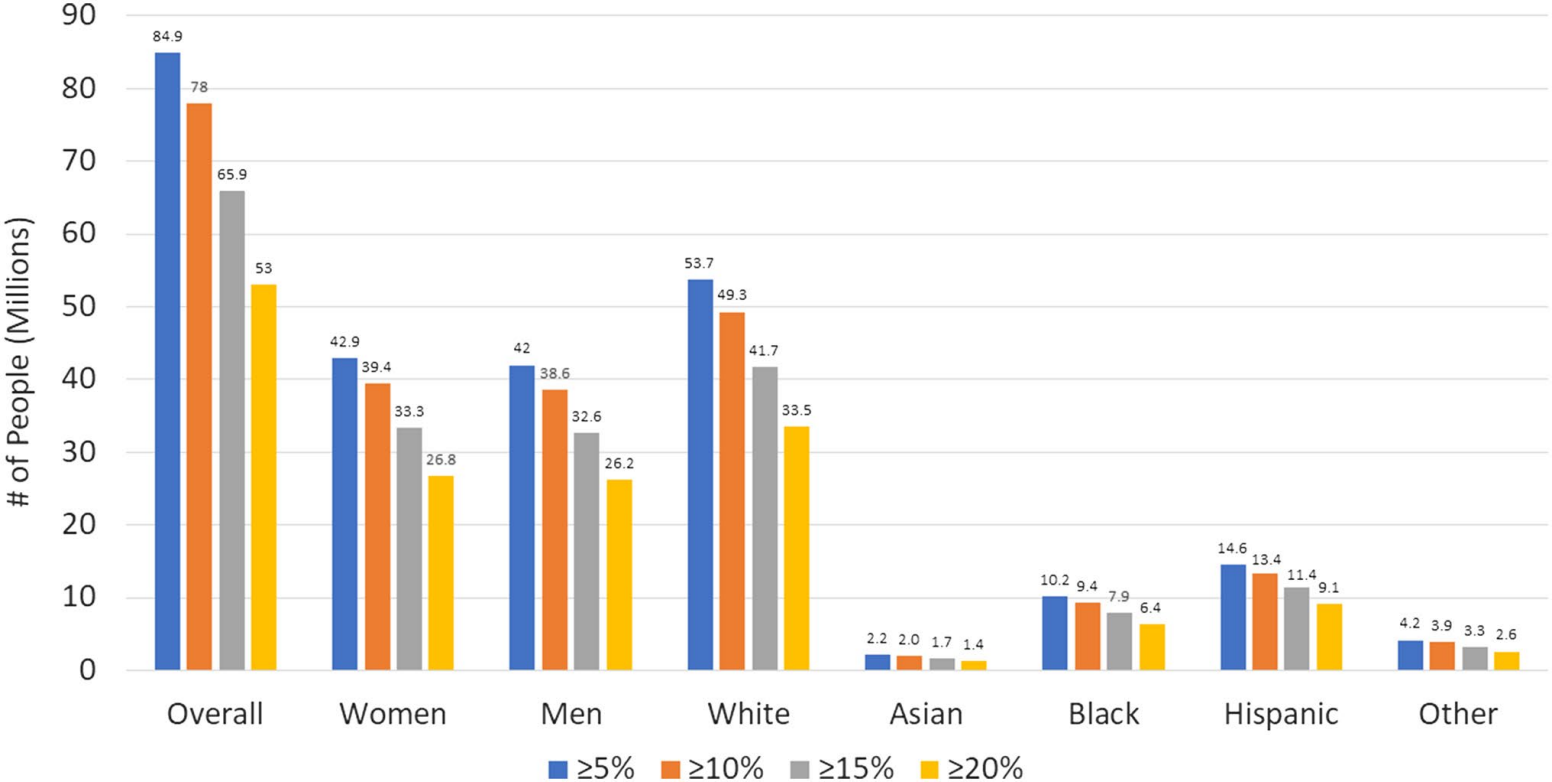
Number of US adults (millions) estimated to have ≥ 5, ≥ 10, ≥ 15%, and ≥ 20% body weight reductions from tirzepatide 15 mg

Figure 2, the Graphical Abstract, and the Supplementary Table 1 showed the estimated number of US adults from our SURMOUNT-1 identified persons we predict would have ≥ 5%, ≥ 10%, ≥ 15%, and ≥ 20% weight reductions from tirzepatide 15 mg treatment. Based on the SURMOUNT-1 trial that showed 90.9%, 83.5%, 70.6%, and 56.7% of tirzepatide treated persons had ≥ 5%, ≥ 10%, ≥ 15%, and ≥ 20% weight reductions, we estimated this to project to 84.9 M, 78.0 M, 65.9 M, and 53.0 M persons among our eligible sample of US adults, respectively. Even if we adjusted for placebo effects, 50.1 M persons would be expected to have ≥ 20% weight reductions, and 57.7 M would have ≥ 15% weight reductions. The number of participants with each degree of weight reduction was similar between males and females, although White persons accounted for the greatest number of persons showing benefit from tirzepatide among our NHANES sample.

**Fig. 3:**
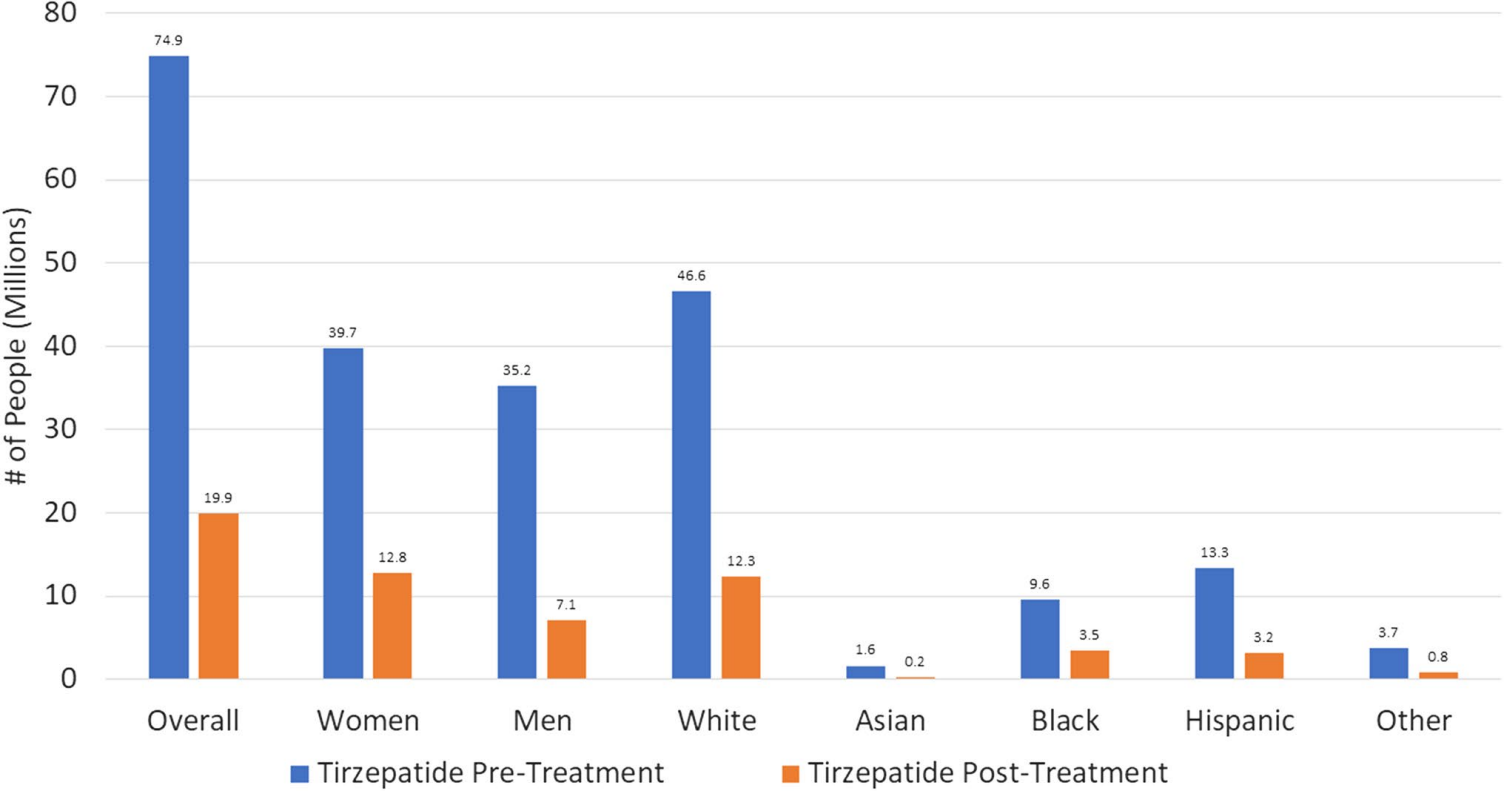
US adults with obesity, in millions before and after tirzepatide treatment based on observed weight changes

Among our study sample, “treatment” with tirzepatide 15 mg weekly we projected a reduction in obesity prevalence of 58.8%, which translated to 55.0 M fewer persons with obesity; among these, 34.0 M transitioned to the overweight category and 34.0 M transitioned to the normal weight category (Supplementary Table 2, Graphical Abstract; Fig. 3). Analyses by sex showed similar number of women (26.9 M) and men (28.1 M) no longer with obesity after tirzepatide “treatment.” Also, again with the largest proportion of our NHANES projected population being White persons, this group had the greatest number of persons no longer obese after tirzepatide treatment.

Among our sample, 3500 persons (83.1 M) did not have previous CVD where we calculated the 10-year CVD risk before and after “treatment” as shown in Table 2 and the Graphical Abstract along with the corresponding numbers (sample and weighted) of estimated CVD events, with the difference reflecting the “preventable” CVD events. The average risk for our sample pre-treatment was 10.14% and was lower in females (7.63%) than in males (12.68%), with the highest risk seen in White persons (11.62%). When we applied the risk factor changes in SURMOUNT-1 based in BMI and systolic blood pressure changes, we observed an overall risk reduction of 2.39% (indicating a number needed to treat of 41.84) and a relative risk reduction of 23.57%. The greatest absolute risk reduction was observed in males (2.95%) and White persons (2.72%). The difference between our estimate of 8.52 M events without treatment with tirzepatide and 6.51 M events with treatment yields 2.01 preventable CVD events, with most of these events among males (1.23 M) and among White persons (1.45 M). If we performed a sensitivity analysis (results not shown) using the laboratory-based equations based on total and HDL-C (with their respective effects from tirzepatide treatment in the SURMOUNT-1 trial) instead of BMI, we obtained pre-treatment and post-treatment CVD risks were 7.62% and 5.79%, respectively. This would yield an absolute risk reduction of 1.83% (and a relative risk reduction of 24.0%), with an estimated 1.47 M preventable CVD events. We found males to have a greater absolute risk reduction (2.15% and1.51%, respectively). White persons also had a greater absolute risk reduction (2.05%) and number of projected preventable CVD events (1.06 M) than other ethnic groups.

**Table 2:** Estimated cardiovascular events and preventable events, based on BMI parameters

	*n* (M)	CVD risk pre-treatment (%)	CVD risk post-treatment (%)	Difference	CVD events pre	CVD events post	Difference
Overall	3500 (84.1 M)	10.14	7.75	2.39%	355 (8.52 M)	271 (6.51 M)	84 (2.01 M)
Female	1916 (42.3 M)	7.63	5.80	1.83%	146 (3.23 M)	111 (2.45 M)	35 (0.77 M)
Male	1584 (41.7 M)	12.68	9.73	2.95%	201 (5.29 M)	154 (4.06 M)	47 (1.23 M)
White	1191 (53.3 M)	11.62	8.90	2.72%	138 (6.19 M)	106 (4.74 M)	32 (1.45 M)
Asian	210 (2.2 M)	7.07	5.39	1.68%	15 (0.15 M)	11 (0.12 M)	4 (0.036 M)
Black	833 (9.8 M)	9.00	6.87	2.13%	75 (0.88 M)	57 (0.67 M)	18 (0.21 M)
Hispanic	1089 (14.8 M)	6.94	5.27	1.67%	76 (1.03 M)	57 (0.78 M)	19 (0.25 M)
Other	177 (4.0 M)	6.65	5.05	1.60%	12 (0.27 M)	9 (0.21 M)	3 (0.064 M)

Estimates combining strata may not total overall due to rounding error. Estimates are based on BMI/non-laboratory Framingham risk algorithms. Sample sizes (*n*) presented with weighted population in millions (M)

## Discussion

We estimate 93 million US adults with overweight and obesity would be potentially eligible for tirzepatide for weight loss based on SURMOUNT-1 eligibility criteria, and of these, 85.9, 78.0, 65.9, and 53.0 million people would show ≥ 5%, ≥ 10%, ≥ 15%, or ≥ 20% weight reductions, respectively. Over half (58.8%) of those initially with obesity would no longer have obesity after treatment with 15 mg tirzepatide. Importantly, we project up to 2 million CV events could be prevented over a 10-years of treatment (among those without prior cardiovascular disease), corresponding to a 2.39% absolute and 23.57% relative risk reduction. Moreover, a cardiovascular event could be expected to be prevented for every 42 eligible persons treated with tirzepatide 15 mg for 10 years. We believe this results to have significant implications on the US population-wide impact of tirzepatide 15 mg therapy for persons with overweight or obesity as well as for ongoing CVD outcomes trials involving tirzepatide.

We also project > 20% weight losses will be observed each in more than 25 million females and males with corresponding reductions in obesity prevalences. Given the US population distribution, the greatest number of persons with these benefits will occur in White, followed by Black, Hispanic, and Asian persons. The greatest number of preventable CVD events are predicted to occur in males, given their greater baseline CVD risk, compared to females, as well as in White persons given their greater population size and baseline risk among our US sample, compared to other race/ethnic groups.

GLP1-RA as well as newer GIP-GLP1-RA are of great interest to treat diabetes and obesity. Since the US Food and Drug Administration (FDA) approval of liraglutide 3.0 mg for obesity [14], subcutaneous semaglutide 2.4 mg was later approved for obesity [15] and most recently tirzepatide also received approval for obesity treatment [16]. It has been previously reported that GLP1-RA therapy reduces weight more in those without diabetes (but with overweight or obesity) than among those with diabetes [17]. Daily liraglutide (3.0 mg) for persons without DM, but with obesity, showed in the SCALE study an average weight loss of 8%, with 63%, 33%, and 14% losing at ≥ 5%, > 10%, and > 15% of body weight, respectively [18]. Using similar BMI cutpoints for tirzepatide 15 mg in the SURMOUNT-1 trial, there was an overall 22.1 kg or 20.9% weight loss, with 90.9% and 83.5% showing weight reductions of at least 5% and 10%, respectively [11]. Most recently, oral GLP1-RA therapies, such as semaglutide 50 mg in the OASIS 1 trial, showed significant weight reductions averaging 15.1% [19], similar to that achieved in the earlier STEP 1 trial [9] with injectable semaglutide. Finally, early studies of the oral non-peptide GLP-1 receptor agonist orforglipron [20], as well as newer triple hormone agonists such as retatrutide [21], are showing benefits of significant weight loss efficacy.

Both GLP1-RA and GIP-GLP-1 RA therapies have also demonstrated improvements in cardiometabolic risk factors, making them promising targets for cardiovascular risk reduction. Liraglutide 3.0 mg daily in the SCALE study showed systolic and diastolic blood pressure reductions of 4.2 and 2.6 mmHg, respectively, and reductions in LDL-cholesterol and triglycerides of 3.0% and 13.3%, respectively, with increases in HDL-cholesterol of 2.3% [18]. In SURMOUNT-1 involving tirzepatide, there were overall (pooled treatment groups) reductions of 15% in body weight, SBP of 7.2 mmHg, total cholesterol of 4.8%, and LDL-cholesterol of 5.8%, increases in HDL-cholesterol of 8.0%, and reductions in triglycerides of 24.8% [11]. Most recently, the SELECT trial involving subcutaneous semaglutide 2.4 mg in overweight or obese persons with pre-existing CVD demonstrated a 20% relative risk reduction in cardiovascular outcomes [22]. Of note, subcutaneous semaglutide 2.4 mg most recently got the additional FDA indication for cardiovascular risk reduction in persons with CVD and overweight/obesity [23]. The SURMOUNT-MMO trial involving tirzepatide [12] will further inform on the role of these newer obesity therapies in reducing CVD outcomes in adults with overweight and obesity with and without prior CVD, and most importantly, confirming in the clinical trial setting whether our projected risk reductions in the present paper hold.

Of interest is the degree of weight loss required to have CVD benefits. Our study projects a 24% relative risk reduction for CVD events based on tirzepatide’s overall > 20% weight reduction (with 84% of patients showing > 10% weight reductions). This compares favorably a post hoc analysis of the Look AHEAD trial data, where the subgroup of subjects among both intervention and control groups combined who lost at least 10% of their body weight in the first year of the study had a 21% lower risk of the primary composite CVD outcome over 10 years of follow-up (realizing, however, the original randomized study did not meet the primary outcome) [24].

There are, however, few data on the population-wide impact of these therapies in populations with overweight or obesity, while their eligibility and impact on CVD events have been previously studied among persons with diabetes. Arnold et al. showed in the US Diabetes Collaborative Registry [25] among 182,525 patients with diabetes derived from 313 multispecialty practices, 48% of patients to fit LEADER eligibility criteria, and estimating 247 MIs and 329 CV deaths per year of treatment could be prevented. We also estimated liraglutide eligibility and potential preventable events among US NHANES adults with diabetes, estimating 15.4% (4.2 million) of those with diabetes to fit LEADER eligibility criteria and from LEADER trial CVD outcome risk reductions observed, 21,209 primary composite CVD events could be prevented annually [26]. Moreover, in a recent analysis of NHANES [27], 51.1% of US adults were estimated to meet the FDA eligibility criteria for tirzepatide with Black (56.6%) and Hispanic adults (55.0%) most often qualifying. Most recently, we also examined the US population impact of subcutaneous semaglutide 2.4 mg, identifying 93 million US adults with overweight or obesity to be eligible based on STEP 1 trial eligibility criteria, with up to a 1.8% absolute (17.8% relative) CVD risk reduction projected that translates to an estimated 1.5 million preventable CVD events over 10 years [28]. The current study provides further data supporting the potential impact of tirzepatide-eligible US adults on obesity prevalence and CVD risk reduction.

Our study has several strengths, limitations, and assumptions. Our study of the NHANES cohort of US adults utilized sample weighting which allows for us to derive US population estimates of SURMOUNT-1 eligible US adults, as well as to estimate how many CVD events could be prevented from its use in the ethnically diverse US population. While NHANES does rely on self-reported measures such as CVD history and cigarette smoking, previous reports have confirmed the reliability of such self-report information [29]. Importantly, the well-known cardiovascular risk factors, namely, weight (for calculation of body mass index), blood pressure, and lipids, were all measured in NHANES participants.

In addition, our simulated impact of the SURMOUNT-1 trial results on our US population sample is not without limitations, most notably assuming the impact of weight reduction and cardiovascular risk factor changes will be similar in our sample as compared to the SURMOUNT-1 sample, as well as the projection of risk factor changes to CVD event reduction can be estimated by the CVD risk algorithms we utilized. Importantly, while NHANES is largely representative of the US population and forms the basis for reported US prevalence data on cardiovascular disease and its risk factors [30], it does not include institutionalized individuals and being a volunteer sample will also lack homeless and other underrepresented persons.

There are also differences between our participants and the SURMOUNT-1 clinical trial participants given the latter was an international trial (e.g., in sex/ethnicity distribution and the prevalence of certain comorbidities) that may affect actual CVD risk, and therefore, the observed effects on weight and cardiovascular risk factors seen in SURMOUNT-1 may not necessarily be translatable to our NHANES sample. For instance, with our sample being slightly older and with a higher proportion men as well as higher average cholesterol and systolic blood pressure levels would result in higher baseline CVD risk (although higher HDL-C levels would result in lower risk), our projections of CVD risk and preventable CVD events may be different than would be the case if our sample was more comparable to that of the SURMOUNT-1 trial.

We are also assuming treatment effects applied to our cohort are the same as in the SURMOUNT-1 trial which was of course a select clinical trial sample and not necessarily fully translatable to real-world populations such as NHANES, thus requiring validation from CVD outcomes trials such as SURMOUNT-MMO [12]. However, such clinical trials, rather forming the basis for treatment indications and guidelines, often have select eligibility criteria along with intensive efforts to ensure adherence to study medication, thus are also not fully translatable to real-world clinic populations.

Also, our estimates are based on the effects seen from the 15-mg dosage in SURMOUNT-1 and suggests the potential impact if all eligible patients were to be on this dosage of the drug; however, it is realized some patients may not achieve this dosage due to side effects or other reasons, and thus, our projections would be less than what we estimated. Our estimates also assume long-term safety and adherence to the therapy, and such information both on long-term safety and adherence are limited. Moreover, since sex and ethnic-specific effects of SURMOUNT-1 are not available, we utilized the overall trial effects for sex and ethnic-specific analyses, and if there were differential effects of tirzepatide by sex or ethnicity, our sex and ethnic-specific projections may be less precisely estimated.

Finally, we have applied the treatment effects seen from the tirzepatide group in the SURMOUNT-1 trial and we cannot be certain whether the preventable CVD events we estimate would be exclusively the result of tirzepatide. Also, since we do not have actual event data resulting from tirzepatide since the CVD outcomes trial is still ongoing, we have used the Framingham Risk Scores to estimate the number of CVD events that can be expected to occur over a defined period of time (e.g., 10 years). Our Framingham risk algorithms, like newer scores such as the Pooled Cohort equations, do not include all possible factors (e.g., family history or other “risk enhancing” factors) that could affect CVD risk. Two important advantages of the Framingham total CVD risk scores [13] we have used are that we are able to include persons beginning at age 30 as well as estimate the prediction of total CVD events (not just myocardial infarction and stroke, but also angina, heart failure and peripheral arterial disease).

## Conclusions

We have estimated that 93.4 million US adults with overweight or obesity could potentially benefit from tirzepatide treatment for long-term weight management. Such treatment at 15 mg dosages could reduce by over 50% the number of persons with obesity, as well as prevent up to 2 million CVD events if on treatment for 10 years. This includes important reductions in obesity prevalence and preventable CVD events in both males and females and the predominant US race/ethnic groups. These results have significant implications for reducing obesity and its related CVD comorbidities as well as associated healthcare costs. Recently reported and ongoing cardiovascular outcomes therapies of newer obesity therapies [11, 22] will document true efficacy of these agents in improving CVD outcomes in persons who are overweight or with obesity. Such outcome data are needed to provide validation for our projected CVD risk reductions and estimated preventable events.

## Electronic supplementary material

Below is the link to the electronic supplementary material.

Supplementary file1 (DOCX 20 KB)

## Data Availability

Data used in this study are publicly available from the National Health and Nutrition Examination Survey: https://www.cdc.gov/nchs/nhanes/index.htm

## Appendix.

Equations used for obtaining CVD 10-year risk, based on the Cox model (based on D’Agostino RB et al. [12]).

BMI-based calculations:

Women:

$$\begin{array}{l}\text{SBPBeta for Treatment}=2.88267;\text{SBPBeta for Non}-\text{Treatment}=2.81291;\\ \text{RiskPart}=2.72107*\text{log}\left(\text{Age}\right)+0.51125*\text{log}\left(\text{BMI}\right)+\text{SBPBeta}*\text{log}\left(\text{SBP}\right)+0.61868*\text{Smoker}+0.77763*\text{Diabetes}\\ \text{CVDRisk}=1-{0.94833}^{\text{exp}\left(\text{RiskPart}-26.0145\right)}\end{array}$$

Men:

$$\begin{array}{l}\text{SBPBeta for Treatment}=1.92672;\text{SBPBeta for Non}-\text{Treatment}=1.85508;\\ \text{RiskPart}=3.11296*\text{log}\left(\text{Age}\right)+0.79277*\text{log}\left(\text{BMI}\right)+\text{SBPBeta}*\text{log}\left(\text{SBP}\right)+0.70953*\text{Smoker}+0.70953*\text{Diabetes};\\ \text{CVDRisk}=1-{0.88431}^{\text{exp}\left(\text{RiskPart}-23.9388\right)}\end{array}$$

Laboratory-based (cholesterol) calculations:

Women:

$$\begin{array}{l}\text{SBPBeta for Treatment}=2.82263;\text{SBPBeta for Non}-\text{Treatment}=2.76157;\\ \text{RiskPart}=2.32888*\text{log}\left(\text{Age}\right)+1.20904*\text{log}\left(\text{Total Cholesterol}\right)-0.70833*\text{log}\left(\text{HDL}\right)+\text{SBPBeta}*\text{log}\left(\text{SBP}\right)+0.61868*\text{Smoker}+0.77763*\text{Diabetes};\\ \text{CVDRisk}=1-{0.95012}^{\text{exp}\left(\text{RiskPart}-26.1931\right)}\end{array}$$

Men:

$$\begin{array}{l}\text{SBPBeta for Treatment}=1.99881;\text{SBPBeta for Non}-\text{Treatment}=1.93303;\\ \text{RiskPart}=3.06117*\text{log}\left(\text{Age}\right)+1.12370*\text{log}\left(\text{Total Cholesterol}\right)-0.93263*\text{log}\left(\text{HDL}\right)+\text{SBPBeta}*\text{log}\left(\text{SBP}\right)+0.70953*\text{Smoker}+0.83160*\text{Diabetes};\\ \text{CVDRisk}=1-{0.88936}^{\text{exp}\left(\text{RiskPartChol}-23.9802\right)}\end{array}$$

## Code Availability

Applicable code may be provided by the authors upon request.

## Declarations

### Ethnics Approval

This study utilized de-identified publicly available data and does not qualify for institutional review board review.
