# Supplementary material for: US Population Eligibility and Estimated Impact of Tirzepatide Treatment on Obesity Prevalence and Cardiovascular Disease Events
Source: Cardiovasc Drugs Ther. 2024 Jun 8;39(4):837–47. doi: 10.1007/s10557-024-07583-z (PMC12296773; doi:10.1007/s10557-024-07583-z)
Supplement: Supplementary file 1 — Supplementary file1 (DOCX 20 KB) [file 10557_2024_7583_MOESM1_ESM.docx]

**Supplementary Table 1. Estimated US Adults with >5, >10, >15%, and ≥20%** **Weight Reductions Based on SURMOUNT-I Trial Placebo, Tirzepatide, and Treatment Group Differences**

| Overall Sample (4015, 93.4M) | Placebo (N=4015) 93.4M | Tirzepatide (N=4015) 93.4M | Tirzepatide-Placebo Difference |
| --- | --- | --- | --- |
| ≥5% | 34.5%/1385/32.2M | 90.9%/3650/84.9M | 56.4%/2264/52.7M |
| ≥10% | 18.8%/755/17.6M | 83.5%/3353/78.0M | 64.7%/2598/60.4M |
| ≥15% | 8.8%/353/8.2M | 70.6%/2835/65.9M | 61.8%/2481/57.7M |
| ≥20% | 3.1%/124/2.9M | 56.7%/2277/53.0M | 53.6%/2152/50.1M |
| Women | Placebo (N=2172) 47.2M | Tirzepatide (N=2172) 47.2M | Tirzepatide-Placebo Difference |
| ≥5% | 34.5%/749/16.2M | 90.9%/1974/42.9M | 56.4%/1225/26.6M |
| ≥10% | 18.8%/408/8.9M | 83.5%/1814/39.4M | 64.7%/1405/30.5M |
| ≥15% | 8.8%/191/4.2M | 70.6%/1533/33.3M | 61.8%/1342/29.2M |
| ≥20% | 3.1%/67/1.5M | 56.7%/1232/26.8M | 53.6%/1164/25.3M |
| Men | Placebo (N=1843) 46.2M | Tirzepatide (N=1843) 46.2M | Tirzepatide-Placebo Difference |
| ≥5% | 34.5%/636/15.9M | 90.9%/1675/42.0M | 56.4%/1039/26.1M |
| ≥10% | 18.8%/346/8.7M | 83.5%/1539/38.6M | 64.7%/1192/29.9M |
| ≥15% | 8.8%/162/4.1M | 70.6%/1301/32.6M | 61.8%/1139/28.6M |
| ≥20% | 3.1%/57/1.4M | 56.7%/1045/26.2M | 53.6%/988/24.8M |
| White | Placebo (N=1405) 59.1M | Tirzepatide (N=1405) 59.1M | Tirzepatide-Placebo Difference |
| ≥5% | 34.5%/485/20.4M | 90.9%/1277/53.7M | 56.4%/792/33.3M |
| ≥10% | 18.8%/264/11.1M | 83.5%/1173/49.3M | 64.7%/909/38.2M |
| ≥15% | 8.8%/124/5.2M | 70.6%/992/41.7M | 61.8%/868/36.5M |
| ≥20% | 3.1%/44/1.8M | 56.7%/797/33.5M | 53.6%/753/31.7M |
| Asian | Placebo (N=233) 2.41M | Tirzepatide (N=233) 2.41M | Tirzepatide-Placebo Difference |
| ≥5% | 34.5%/80/0.8M | 90.9%/212/2.2M | 56.4%/131/1.4M |
| ≥10% | 18.8%/44/0.5M | 83.5%/195/2.0M | 64.7%/151/1.6M |
| ≥15% | 8.8%/21/0.2M | 70.6%/164/1.7M | 61.8%/144/1.5M |
| ≥20% | 3.1%/7/0.07M | 56.7%/132/1.4M | 53.6%/125/1.3M |
| Black | Placebo (N=974) 11.2M | Tirzepatide (N=974) 11.2M | Tirzepatide-Placebo Difference |
| ≥5% | 34.5%/336/3.9M | 90.9%/885/10.2M | 56.4%/549/6.3M |
| ≥10% | 18.8%/183/2.1M | 83.5%/813/9.4M | 64.7%/630/7.3M |
| ≥15% | 8.8%/86/1.0M | 70.6%/688/7.9M | 61.8%/602/6.9M |
| ≥20% | 3.1%/30/0.3M | 56.7%/552/6.4M | 53.6%/522/6.0M |
| Hispanic | Placebo (N=1199) 16.1M | Tirzepatide (N=1199) 16.1M | Tirzepatide-Placebo Difference |
| ≥5% | 34.5%/414/5.6M | 90.9%/1090/14.6M | 56.4%/676/9.1M |
| ≥10% | 18.8%/225/3.0M | 83.5%/1001/13.4M | 64.7%/776/10.4M |
| ≥15% | 8.8%/106/1.4M | 70.6%/846/11.4M | 61.8%/741/1.0M |
| ≥20% | 3.1%/37/0.5M | 56.7%/680/9.1M | 53.6%/643/8.6M |
| Other | Placebo (N=204) 4.62M | Tirzepatide (N=204) 4.62M | Tirzepatide-Placebo Difference |
| ≥5% | 34.5%/70/1.6M | 90.9%/185/4.2M | 56.4%/115/2.6M |
| ≥10% | 18.8%/38/0.9M | 83.5%/170/3.9M | 64.7%/132/3.0M |
| ≥15% | 8.8%/18/0.4M | 70.6%/144/3.3M | 61.8%/126/2.9M |
| ≥20% | 3.1%/6/0.1M | 56.7%/116/2.6M | 53.6%/109/2.5M |

Sample sizes (n) presented with weighted population in millions (M).

**Supplemental Table 2. Proportion and Number of US Adults (n and millions) Pre and Post-Tirzepatide Treatment in Each Weight Category Based on Observed Weight Changes (From SURMOUNT-1 Trial Paper)**

| Overall Sample: (4015, 93.4M) | Pre-Treatment | Post-Treatment | Difference |
| --- | --- | --- | --- |
| Normal (<25) | 0%/0/0.0M | 36.4%/1496/34.0M | +36.4%/+1496/+34.0M |
| Overweight (25 to <30) | 19.9%/818/18.5M | 42.3%/1647/39.5M | +22.4%/+829/+21.0M |
| Obese (≥30) | 80.1%/3197/74.9M | 21.3%/872/19.9M | -58.8%/-2325/-55.0M |
| Women: (2172, 47.2M) | Pre-Treatment | Post-Treatment | Difference |
| Normal (<25) | 0%/0/0.0M | 30.5%/683/14.4M | +30.5%/+683/+14.4M |
| Overweight (25 to <30) | 15.9%/358/7.50M | 42.5%/910/20.0M | +26.6%/+552/+12.5M |
| Obese (≥30) | 84.1%/1814/39.7M | 27.1%/579/12.8M | -57.0%/-1235/-26.9M |
| Men: (1843, 46.2M) | Pre-Treatment | Post-Treatment | Difference |
| Normal (<25) | 0%/0/0.0M | 42.4%/813/19.6M | +42.4%/+813/+19.6M |
| Overweight (25 to <30) | 23.9%/460/11.0M | 42.2%/737/19.5M | +18.3%/+277/+8.50M |
| Obese (≥30) | 76.1%/1383/35.2 M | 15.4%/293/7.12M | -60.7%/-1090/-28.1M |
| White: (1405, 59.1M) | Pre-Treatment | Post-Treatment | Difference |
| Normal (<25) | 0%/0/0.0M | 37.4%/542/22.1M | +37.4%/+542/+22.1M |
| Overweight (25 to <30) | 21.1%/314/12.4M | 41.8%/566/24.7M | +20.7%/+252/+12.3M |
| Obese (≥30) | 78.9%/1091/46.6M | 20.7%/297/12.3M | -58.2%/-794/-34.3M |
| Asian: (233, 2.41M) | Pre-Treatment | Post-Treatment | Difference |
| Normal (<25) | 0%/0/0.0M | 56.4%/135/1.4M | +56.4%/+135/+1.4M |
| Overweight (25 to <30) | 33.0%/84/0.8M | 36.5%/81/0.9M | +3.5%/-3/+0.1M |
| Obese (≥30) | 67.0%/149/1.6M | 7.1%/17/0.2M | -59.9%/-132/-1.4M |
| Black: (974, 11.2M) | Pre-Treatment | Post-Treatment | Difference |
| Normal (<25) | 0%/0/0.0M | 29.6%/310/3.3M | +29.6%/+310/+3.3M |
| Overweight (25 to <30) | 14.5%/159/1.6M | 39.3%/379/4.4M | +24.8%/+220/+2.8M |
| Obese (≥30) | 85.5%/815/9.6M | 31.1%/285/3.5M | -54.4%/-530/-6.1M |
| Hispanic: (1199, 16.1M) | Pre-Treatment | Post-Treatment | Difference |
| Normal (<25) | 0%/0/0.0M | 34.4%/446/5.5M | +34.4%/+446/+5.5M |
| Overweight (25 to <30) | 17.2%/231/2.8M | 45.9%/523/7.4M | +28.7%/+292/+4.6M |
| Obese (≥30) | 82.8%/968/13.3M | 19.7%/230/3.2M | -63.1%/-738/-10.1M |
| Other: (204, 4.62M) | Pre-Treatment | Post-Treatment | Difference |
| Normal (<25) | 0%/0/0.0M | 35.8%/63/1.7M | +35.8%/+63/+1.7M |
| Overweight (25 to <30) | 19.8%/30/1.0M | 46.5%/98/2.2M | +26.7%/+68/+1.2M |
| Obese (≥30) | 80.2%/174/3.7M | 17.7%/43/0.8M | -62.5%/-131/-2.9M |

Sample sizes (n) presented with weighted population in millions (M).
